# 3D-printed titanium implant-coated polydopamine for repairing femoral condyle defects in rabbits

**DOI:** 10.1186/s13018-020-01593-x

**Published:** 2020-03-11

**Authors:** Weiyang Zhong, Jianxiao Li, Chenbo Hu, Zhengxue Quan, Dianming Jiang, Guangbin Huang, Zhigang Wang

**Affiliations:** 1 Department of Orthopedic Surgery, The First Affiliated Hospital of Chongqing Medical University, Chongqing, 400016 China; 2 Department of Orthopedic Surgery, The Third Affiliated Hospital of Chongqing Medical University, Chongqing, 400042 China; 3 Department of Trauma Surgery, Emergency Medical Center of Chongqing, The Affiliated Central Hospital of Chongqing University, Chongqing, 400014 China; 4 Institute of Ultrasound Imaging, The Second Affiliated Hospital of Chongqing Medical University, Chongqing, 400010 China

**Keywords:** 3D-printed porous titanium, Polydopamine, Bone defect

## Abstract

**Background:**

Large segmental bone defects are still one of the challenges for orthopaedic surgeons. Although 3D-printed porous titanium is a potential bone substitute material because of its porous structure simulating natural bone, the titanium surface has low bioactivity, integrates with bone tissue through the simple mechanical interlock. The study aims to investigate the capability and osteogenesis of 3D-printed porous titanium (3D PPT)-coated polydopamine (PDA) for repairing bone defects.

**Methods:**

Fifteen 6-month New Zealand white rabbits were implanted with PDA-3D PPT to repair 6 mm × 10 mm defects on the femoral condyle compared with the group of 3D PPT and comparing with the blank group. After 6 weeks and 12 weeks, micro-CT and histological examination were performed to observe bone growth.

**Results:**

All the PDA-3D PPT group, the 3D PPT group and the blank group recovered in good condition. The images showed that the boundaries between the implant area and the surrounding area were obscure in the three groups. The results of micro-CT demonstrated that at 6 weeks and 12 weeks, the bone volume (BV) values of PDA-3D PPT implants group were significantly higher than those of the 3D PPT implants group and blank group (*P* < 0.05), the BV/tissue volume (TV) and the trabecular number (Tb.N) of PDA-3D PPT implants were significantly higher than those of the 3D PPT group and blank group (*P* < 0.05). The results of un-decalcified bone slicing showed that ore new bone appeared to form around the PDA-3D PPT than that of 3D PPT and blank group. The bone-implant contact (BIC) of PDA-3D PPT was better (*P* < 0.05) than that of 3D PPT group.

**Conclusion:**

PDA-3D PPT could improve the bioactivity and promote the growth and healing of bone tissue and can be a promising repairing material.

## Background

Large segmental bone defects caused by high-energy trauma, infection, bone tumour or congenital malformation are still one of the challenges for orthopaedic surgeons. At present, the most common management strategies involve autologous bone grafts or allogeneic grafts, which have advantages and disadvantages. Autologous bone grafting requires additional surgery and more trauma, which often have complications, such as nerve damage, fractures and infections. Allogeneic bone grafts have the risks of immune diseases and of slowing bone remodelling [1–3]. 3D-printed porous titanium (3D PPT) is a potential bone substitute material because of its porous structure simulating natural bone, which is beneficial to the growth of new bone tissue and provides a new way to treat bone defects [4–8].

However, the titanium surface has low bioactivity, forms a simple mechanical interlock with the bone tissue and cannot integrate with the bone. Dopamine, which contains catecholamine functional groups and lysine end amino groups, can form very strong adhesion dopamine (polydopamine, PDA) films on the surface of glass, metal, ceramics, organic matter and other materials under alkaline conditions. PDA can fix biomolecules on the surface of a modified material [9, 10].

We used a laser three-dimensional forming technology to process titanium powder to prepare 3D PPT coated by PDA, acquiring a good hydrophilic surface to use biochemical surface modification methods to modify the bone-implant surface with a high specific surface area and increasing the mechanical locking of the implant and the bone.

The capacity of the modified 3D PPT to repair femur defects was investigated in vivo, which could provide the experimental basis for clinical application.

## Material and methods

### Preparation of PDA-coated 3D PPT

**Fig. 1:**
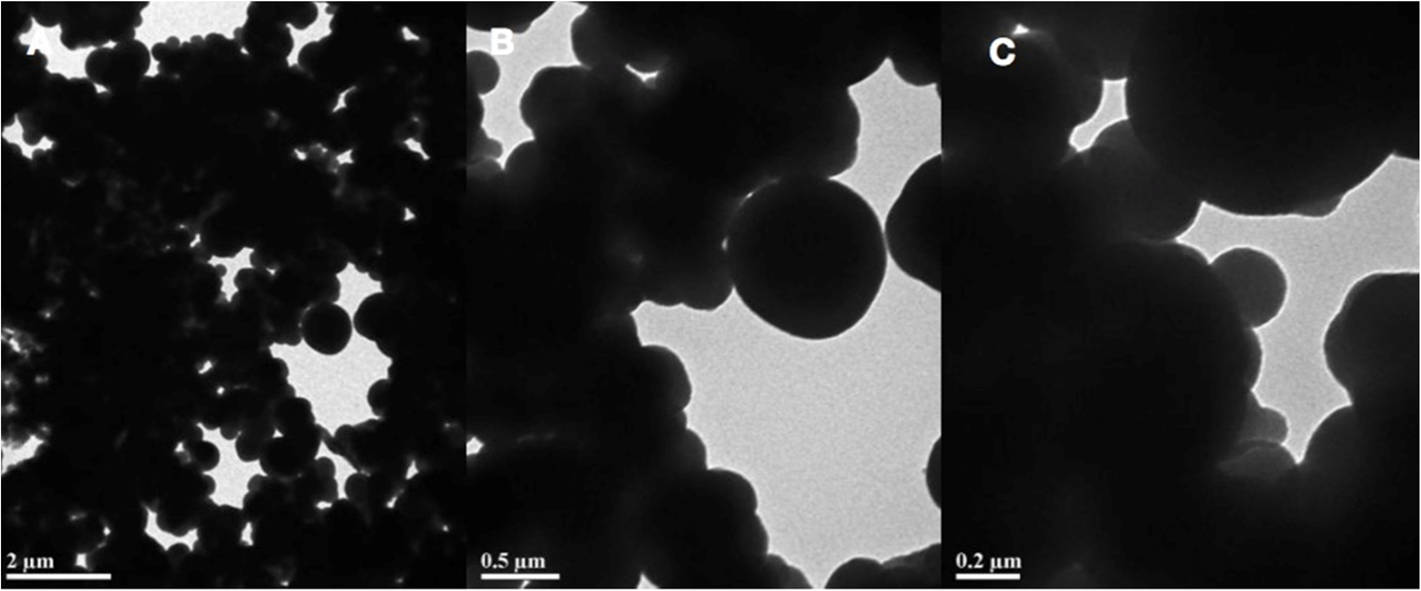
SEM images of PDA. **a** ×2 μm; **b** ×0.5 μm; **c** ×0.2 μm

**Fig. 2:**
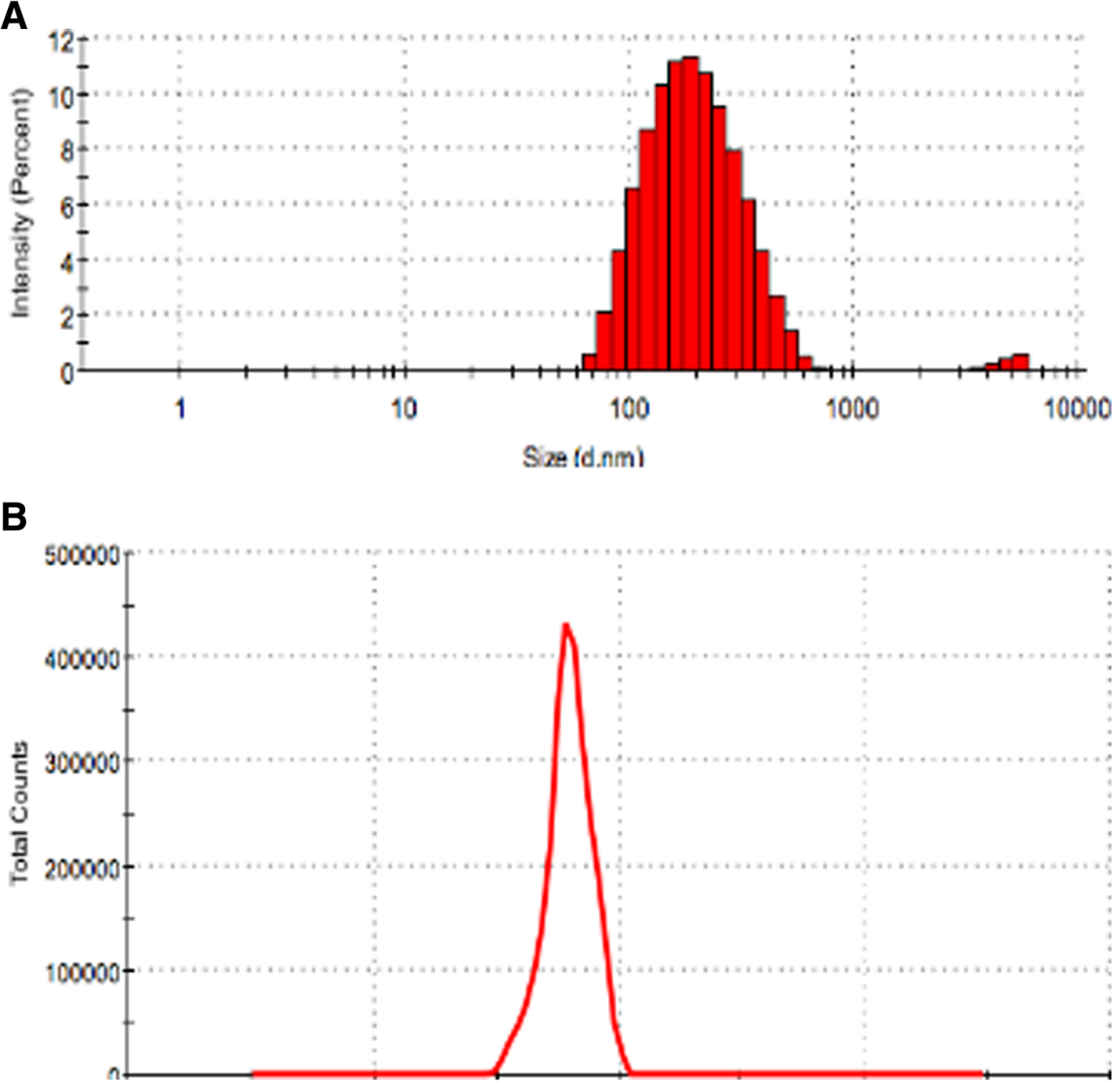
Particle size and zeta potential of PDA. **a** Particle size; **b** zeta potential

The preparation of the PDA coating was performed according to the described method [10, 11] (Figs. 1 and 2). Titanium alloy (Ti6AL4V) implants prepared using selective laser melting and containing a three-dimensional implant architecture were designed as a cylindrical type (6.0 mm diameter × 10 mm long) and used for implantation.

### Material surface characterization

**Fig. 3:**
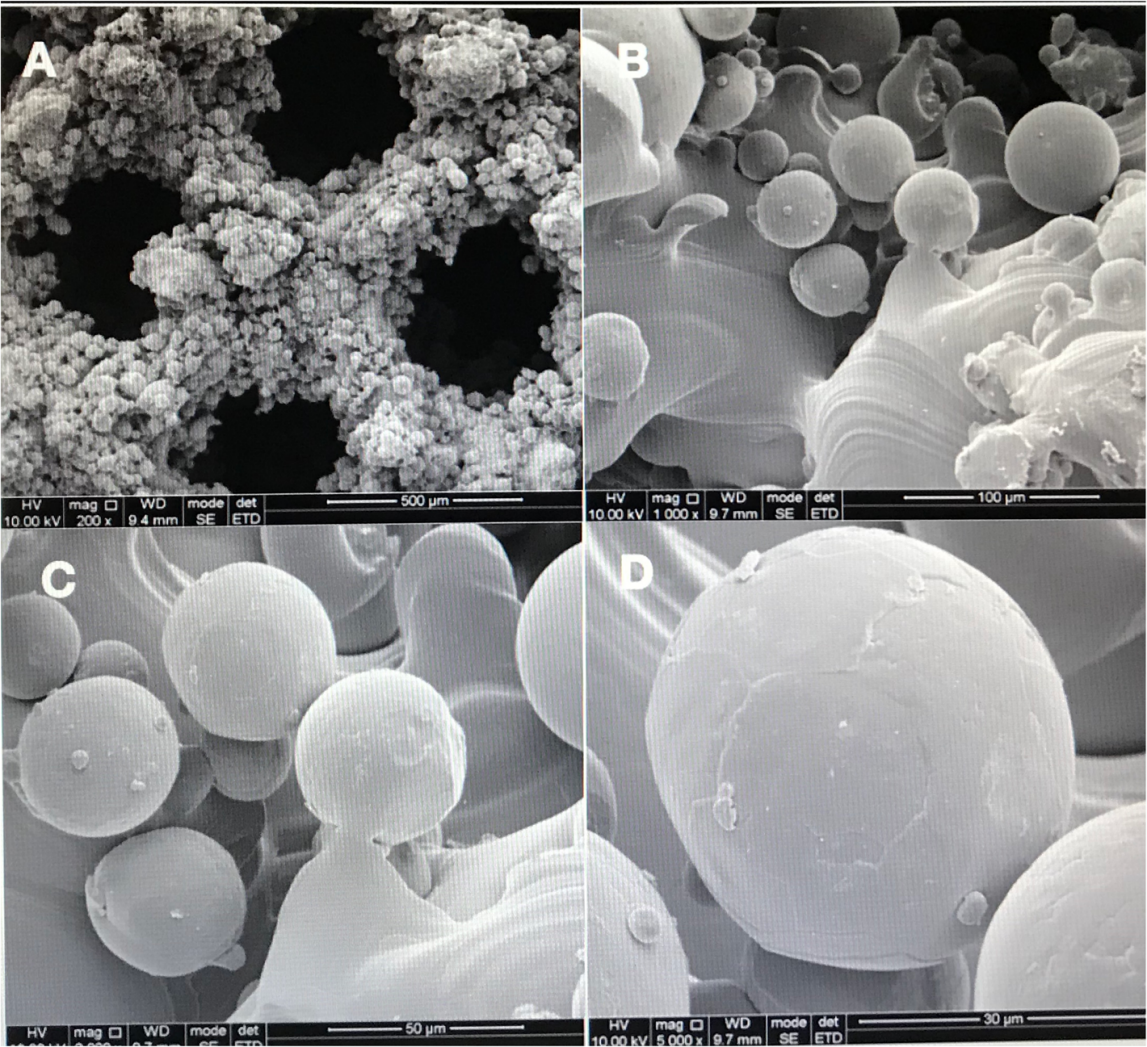
SEM images of PDA-3D PPT. **a** ×200 μm; **b** ×100 μm; **c** ×50 μm; **d** ×30 μm

**Fig. 4:**
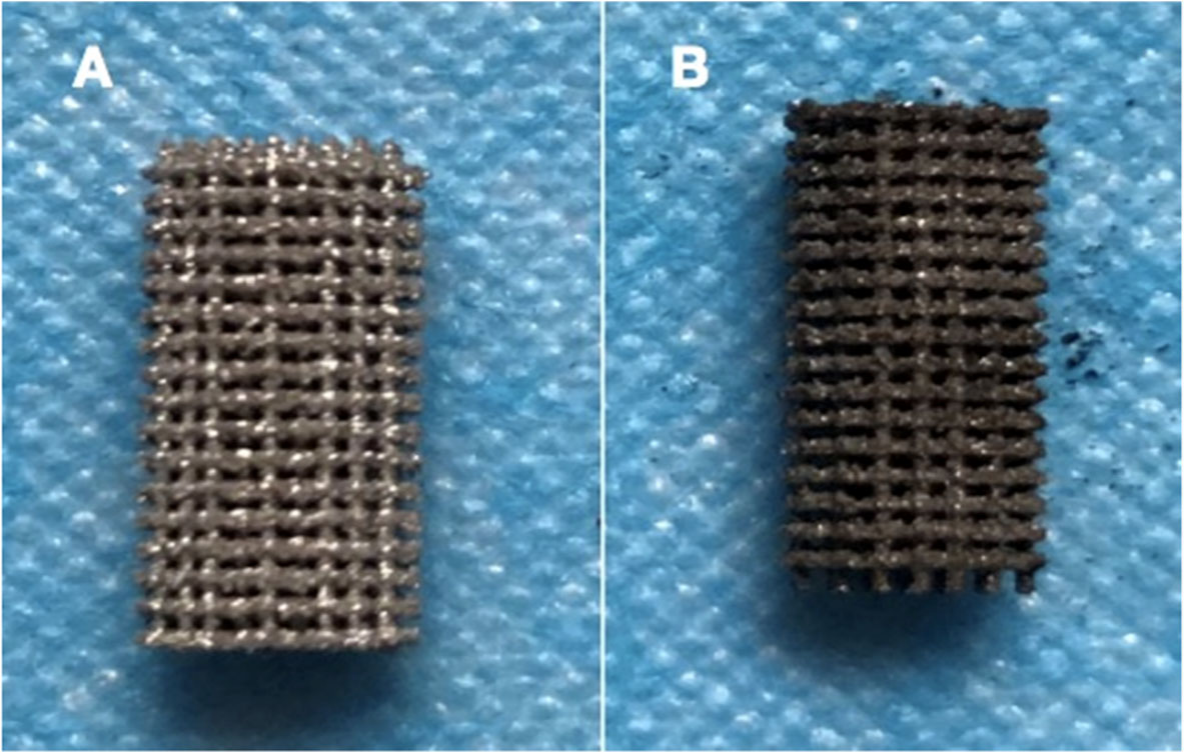
Images of 3D PPT and PDA-3D PPT. **a** 3D PPT; **b** PDA-3D PPT

The surface morphology of 3D PPT implant-coated polydopamine and 3D PPT implants was observed using scanning electron microscopy (SEM; JSM-7500; JEOL, Tokyo, Japan) to confirm the presence of the PDA coating on the surface of the 3D PPT (Figs. 3 and 4).

### Experimental animals and surgical procedure

All animal experiment protocols were conducted according to the procedures approved by the Animal Care and Experiment Committee of The First Affiliated Hospital of Chongqing Medical University and were approved by the animal ethics committee of The First Affiliated Hospital of Chongqing Medical University (no: 20187801). Fifteen adult, male New Zealand white rabbits weighing 2.0–3.0 kg were applied in our study and were randomly divided into three groups (***n*** = 5 each). The one-side femur of each rabbit was used as the surgical site. General anaesthesia was induced by intravenous injection (1.0 mL/kg) with a 3% pentobarbital sodium solution (Sigma-Aldrich Co.). After anaesthetisation, the surgical area was shaved and disinfected with iodine. After a longitudinal 3.5 cm lateral incision was made, the patella was dislocated medially, and the femoral groove was exposed. With the knees flexed, the bone defects (6 mm width × 10 mm depth) were manually created in the femoral groove with a bone driller (4 mm width × 2 mm thickness). Then, the 3D PPT and PDA-3D PPT implants were installed in the defects and the blank group was without any implant. The incisions were sutured in layers, and the lateral ligaments were sutured tightly to avoid patellar dislocation. Penicillin sodium (Southwest Pharmaceutical Co., Ltd., Chongqing, PRC; 200,000 IU/kg/day, intramuscular injection) was administered for 3 days postoperatively. The rabbits were kept in separate cages and were allowed full activity after surgery.

### Micro-CT measurements and histological evaluations

Micro-CT scanning and histological staining were applied to observe bone formation around the implants at 6 and 12 weeks postoperatively.

### Statistical analysis

Statistical analysis was performed using the Statistic Analysis System (SAS Institute Inc., Cary, NC, USA). Quantitative variables were described as the mean ± SD. Student’s ***t*** test was used for the statistical analysis of differences in mean values, and the chi-squared test was used for categorical data. Differences with a ***P*** value < 0.05 were considered.

## Results

After the implantation of the material, the general condition of the rabbits was good. The incisions had different degrees of swelling, but there were no infections. The rabbits were sacrificed 6 weeks and 12 weeks after the operation, and the specimens were removed. The new bone tissues were filled with the experimental bones.

Micro-CT revealed fibrous tissue growth in the bone defect area 6 weeks after surgery. Twelve weeks after surgery, the three groups of specimens showed different degrees of periosteal reaction in the bone defect area and the implant material, and a small amount of callus formation was observed. At 12 weeks postoperatively, the specimens were taken for micro-CT examination. The formation of calli was observed in all groups. The density of PDA-3D PPT implanted in the experimental group was higher than that in the 3D PPT group and blank group of the surrounding host bone, and more bone callus was formed. The boundary between the bone and its surrounding was blurred, and the porous area was filled.

**Fig. 5:**
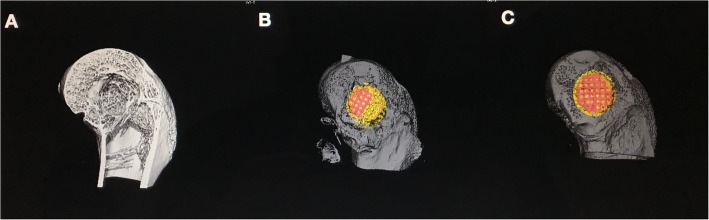
Micro-CT 3D reconstruction images on the femoral defect. **a** Blank; **b** 3D PPT; **c** PDA-3D PPT

**Fig. 6:**
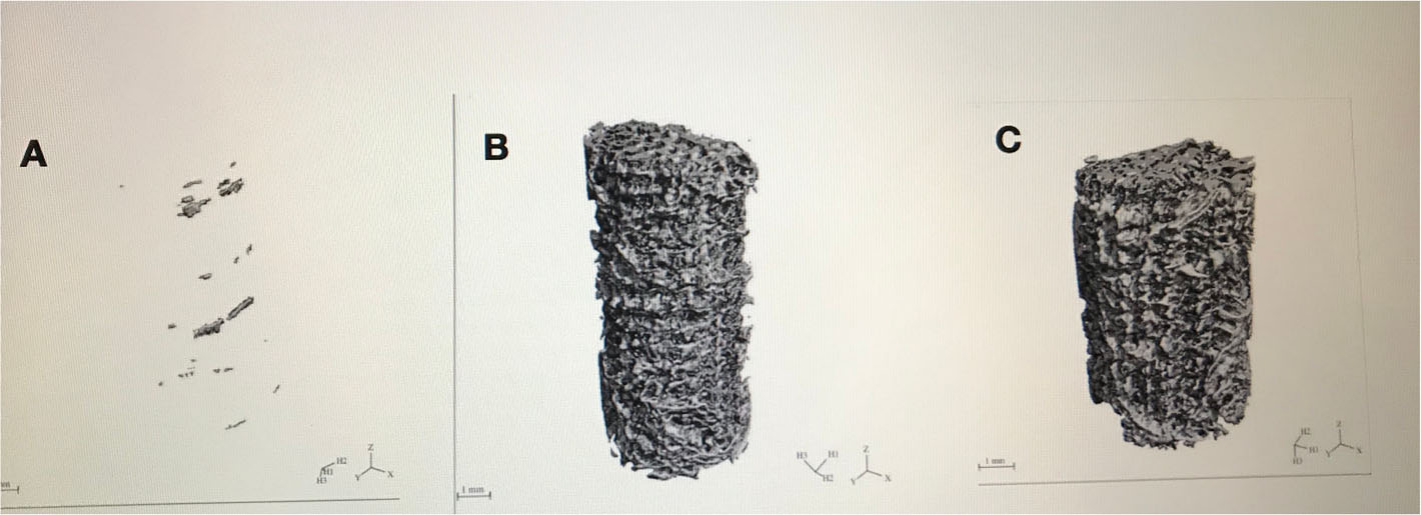
Micro-CT 3D reconstruction images of 3D PPT and PDA-3D PPT of peri-implant bone with 1 mm. **a** Blank; **b** 3D PPT; **c** PDA-3D PPT

**Fig. 7:**
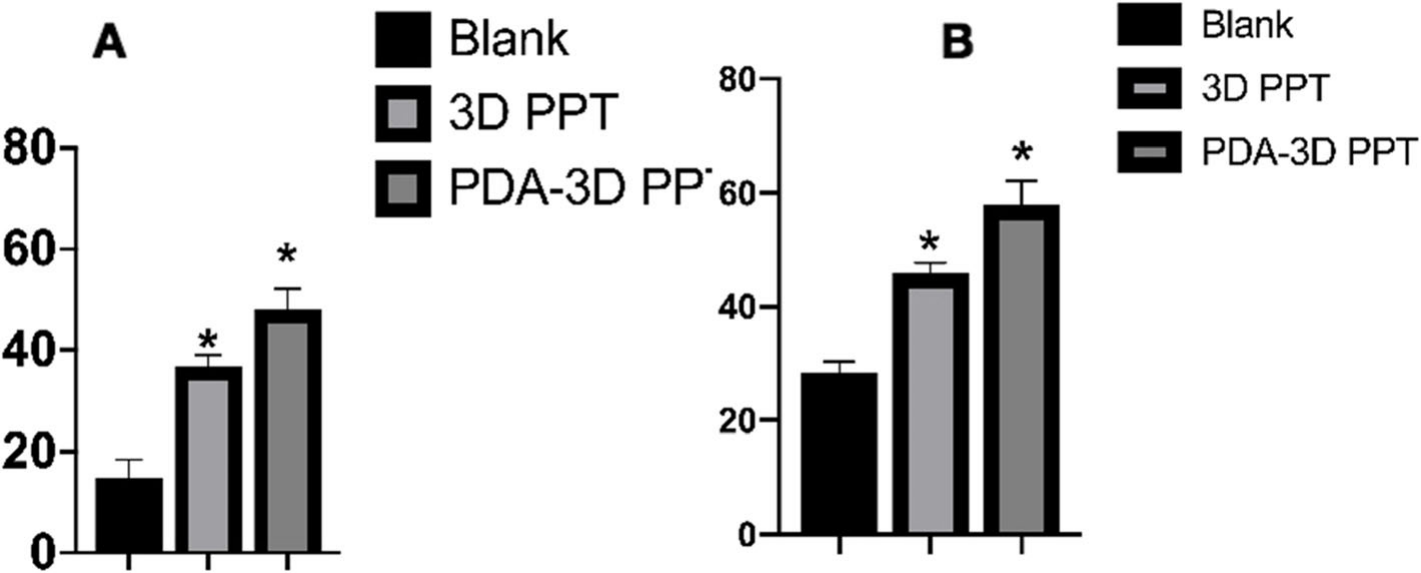
BV shows significant differences in both groups. **a** 6 weeks; **b** 12 weeks

**Fig. 8:**
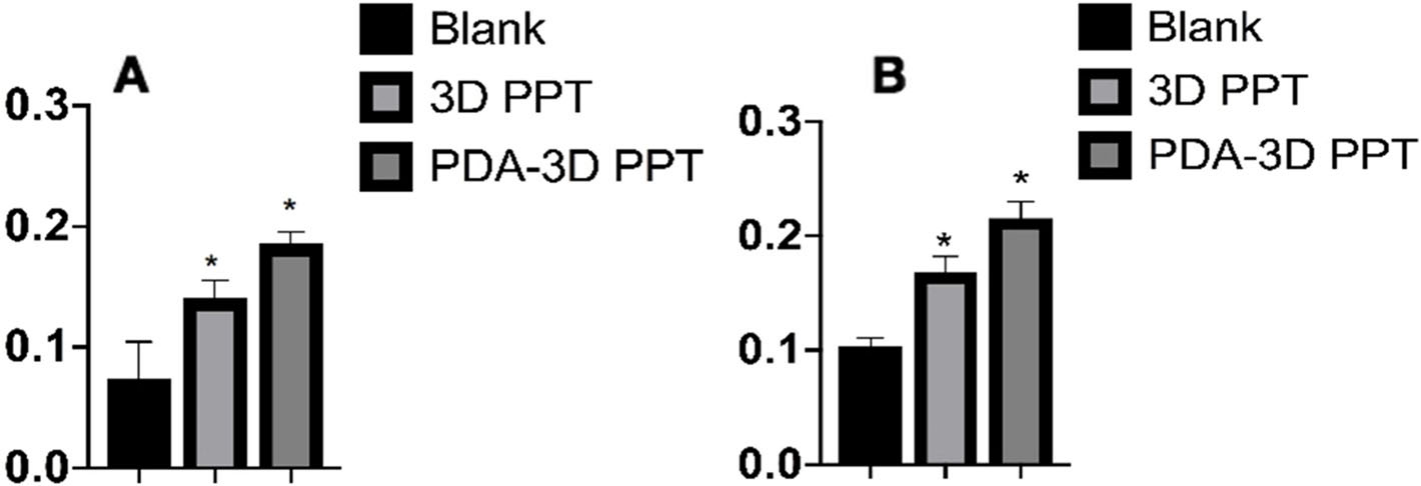
The BV density values (BV/TV%) are significantly different. **a** 6 weeks; **b** 12 weeks

**Fig. 9:**
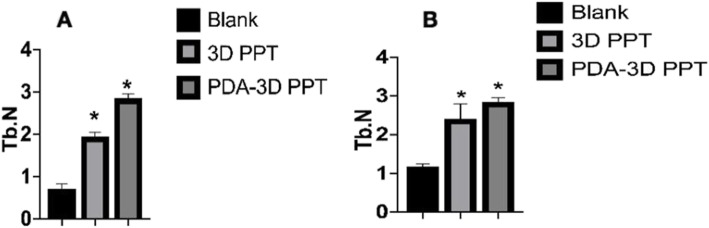
The Tb.N are significantly different. **a** 6 weeks; **b** 12 weeks

The micro-CT 3D reconstruction images showed that more new bone appeared to form within the VOI around the PDA-3D PPT implants than that detected in other groups (Figs. 5 and 6). At 6 weeks and 12 weeks, the bone volume (BV) values of PDA-3D PPT implant group were significantly higher than those of the 3D PPT implants group and blank group (*P* < 0.05) (Fig. 7). To quantitatively analyse the formation of new bone, the BV/tissue volume(TV) and the trabecular number (Tb.N) of each group were determined by micro-CT analysis. As shown, the BV/TV values and Tb.N of PDA-3D PPT implants were significantly higher than those of the 3D PPT group and blank group (*P* < 0.05) (Figs. 8 and 9).

**Fig. 10:**
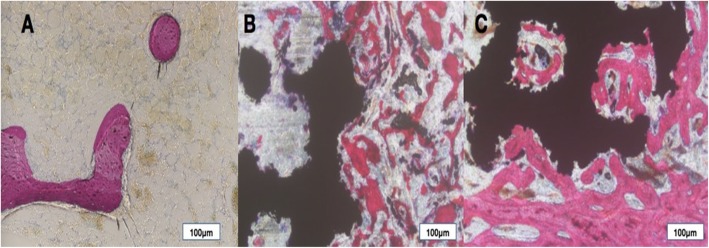
Histological analysis (H&E staining) of femoral condyle defects at 12 weeks after surgery. **a** Blank; **b** 3D PPT; **c** PDA-3D PPT

**Fig. 11:**
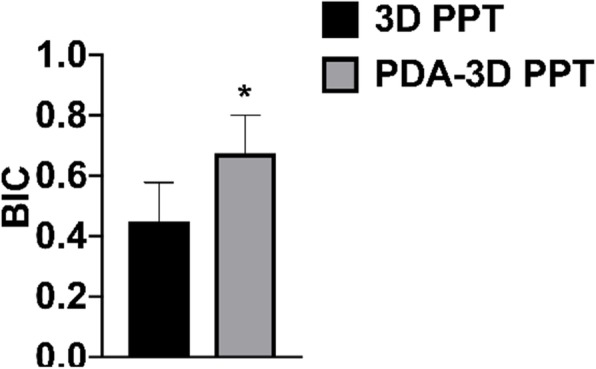
BIC of 3D PPT and PDA-3D PPT

At 12 weeks after surgery, the PDA-3D PPT was tightly implanted with the surrounding bone tissue and was closely combined, and there was no obvious inflammatory cell infiltration or interfiber tissue. There was no obvious bone resorption or osteolysis in the implant material area. More new bone appeared to form around the PDA-3D PPT than that of 3D PPT and blank group (Fig. 10). The bone-implant contact (BIC) of PDA-3D PPT was better (*P* < 0.05) than that of the 3D PPT group (Fig. 11).

## Discussion

Bone defects caused by trauma, infection, and tumour resection are common and seriously endanger bone healing [1–4]. Currently, porous titanium implants based on 3D printing have become a bone substitute because of their porous property and the open porous structure facilitates the transport of blood and nutrients in the implant, promoting tissue regeneration and reconstruction and speeds up the repair process [5–10]. However, the titanium surface has low bioactivity, forms a simple mechanical interlock with the bone tissue, and does not induce bone integration. The ideal goal of implant surface modification could achieve strong osseointegration and improve long-term stability and the osseointegration depends on direct interaction between the bone tissue and biomaterial surface. The 3D PPT coating is one of the most common methods for surface modification of biomaterials and can improve the process of osseointegration [11–16].

The PDA film was performed by dopamine can produce a polymerized layer on almost any shape and material type by oxidative self-polymerization under alkaline conditions. As shown in Figs. 1, 2, 3, 4, the mean diameter of PDA particle was 100–300 nm and the SEM images demonstrated significant numbers of spherical particles formed on the surface of the 3D PPT which increased greatly the surface area. The previous studies showed that PDA preparation on substrates can provide many hydroxyl groups(OH–), attracting calcium ions (Ca2+) and subsequently bond with phosphate ions (PO3–)which could improve the bioactivity of 3D PPT, forming a stronger interlock with the bone tissue [17–20].

Direct bone attachment and integration on the implant interface plays a key role in the implant performance. The effects of PDA coatings on osseointegration were further studied in vivo. In our study, micro-CT and histological examinations were conducted to detect new bone formation at the bone-implant interface. We observed that newly formed bone was tightly bound in blank group. Furthermore, the more newly formed bone could be observed at the bone-implant interface of PDA-3D PPT compared with 3D PPT. Quantitative analysis of micro-CT further confirmed that more new bone was formed around PDA-3D PPT than the other two groups. Histological examination and BIC resulted in the same conclusion. The results showed that PDA-3D PPT had a more positive effect on osseointegration in vivo than the 3D PPT.

## Conclusion

In our study, a PDA coating was successfully prepared on the surface of a 3D PPT material. In vivo studies, micro-CT analysis and histological findings revealed that the PDA coating significantly improved the osteogenic induction of PDA-3D PPT implants. In conclusion, PDA-3D PPT improved the osteogenesis-inducing ability of the biomaterials and provided the potential for the surfacemodification of implanted materials.

## Data Availability

The datasets used and/or analyzed during the current study are available from the corresponding author on reasonable request.

## Abbreviations

3D PPT: 3D-printed porous titanium BIC Bone-implant contact BV Bone volume
PDA: Polydopamine
Tb.N: Trabecular number
TV: Tissue volume
